# Elevated Expression of Squamous Cell Carcinoma Antigen (SCCA) Is Associated with Human Breast Carcinoma

**DOI:** 10.1371/journal.pone.0019096

**Published:** 2011-04-19

**Authors:** Joseph M. Catanzaro, Jennifer L. Guerriero, Jingxuan Liu, Erica Ullman, Namratha Sheshadri, John J. Chen, Wei-Xing Zong

**Affiliations:** 1 Department of Molecular Genetics and Microbiology, Stony Brook University, Stony Brook, New York, United States of America; 2 Department of Pathology, Stony Brook University, Stony Brook, New York, United States of America; 3 Department of Preventative Medicine, Stony Brook University, Stony Brook, New York, United States of America; Ludwig-Maximilians University, Germany

## Abstract

Squamous cell carcinoma antigen (SCCA) belongs to the serine protease inhibitor (Serpin) family of proteins. Elevated expression of SCCA has been used as a biomarker for aggressive squamous cell carcinoma (SCC) in cancers of the cervix, lung, head and neck, and liver. However, SCCA expression in breast cancer has not been investigated. Immunohistochemical analysis of SCCA expression was performed on tissue microarrays containing breast tumor tissues (n = 1,360) and normal breast epithelium (n = 124). SCCA expression was scored on a tiered scale (0-3) independently by two evaluators blind to the patient's clinical status. SCCA expression was observed in Grade I (0.3%), Grade II (2.5%), and Grade III (9.4%) breast cancers (p<0.0001). Comparing tissues categorized into the three non-metastatic TNM stages, I-III, SCCA positivity was seen in 2.4% of Stage I cancers, 3.1% of Stage II cancers, and 8.6% of Stage III breast cancers (p = 0.0005). No positive staining was observed in normal/non-neoplastic breast tissue (0 out of 124). SCCA expression also correlated to estrogen receptor/progesterone receptor (ER/PR) double-negative tumors (p = 0.0009). Compared to SCCA-negative patients, SCCA-positive patients had both a worse overall survival and recurrence-free survival (p<0.0001 and p<0.0001, respectively). This study shows that SCCA is associated with both advanced stage and high grade human breast carcinoma, and suggests the necessity to further explore the role of SCCA in breast cancer development and treatment.

## Introduction

Squamous cell carcinoma antigens (SCCA) are members of the serpin family of endogenous serine proteinase inhibitors. The first variant of SCCA, SCCA1, was originally identified in squamous cell carcinoma (SCC) of the uterine cervix [1]. Further studies found that SCCA1 and its isoform, SCCA2, are produced by two tandemly arranged genes located on chromosome 18q21 [2]. SCCA1 and SCCA2 are approximately 98% and 92% homologous at their nucleotide and amino acid levels, respectively. Although SCCA1 and SCCA2 inhibit different classes of proteases, dictated by differences in amino acids located in the reactive site loop (RSL), both isoforms are expressed in stratified squamous epithelia and have been found to be produced in SCCs [3], [4]. High levels of SCCA are often associated with poorly differentiated and advanced metastatic SCCs. In clinical practice, immunohistochemistry on tissue biopsies and ELISA-based detection of circulating SCCA (including both SCCA1 and SCCA2) are currently used as valuable predictors of nodal metastasis, response to treatment, and tumor recurrence in SCCs of the uterine cervix, lung, head and neck, esophagus, and liver [5], [6].

Thus far, elevated levels of SCCA have been seen in cancers of epithelial (cervix, lung, head and neck) and endodermal (liver) origin. However, despite the epithelial origin of breast ductal and lobular carcinomas, there have been no reports correlating SCCA expression to breast cancer. Here, we sought to examine whether SCCA is associated with human breast cancer.

## Results

### Validation of SCCA antibodies

**Figure 1:**
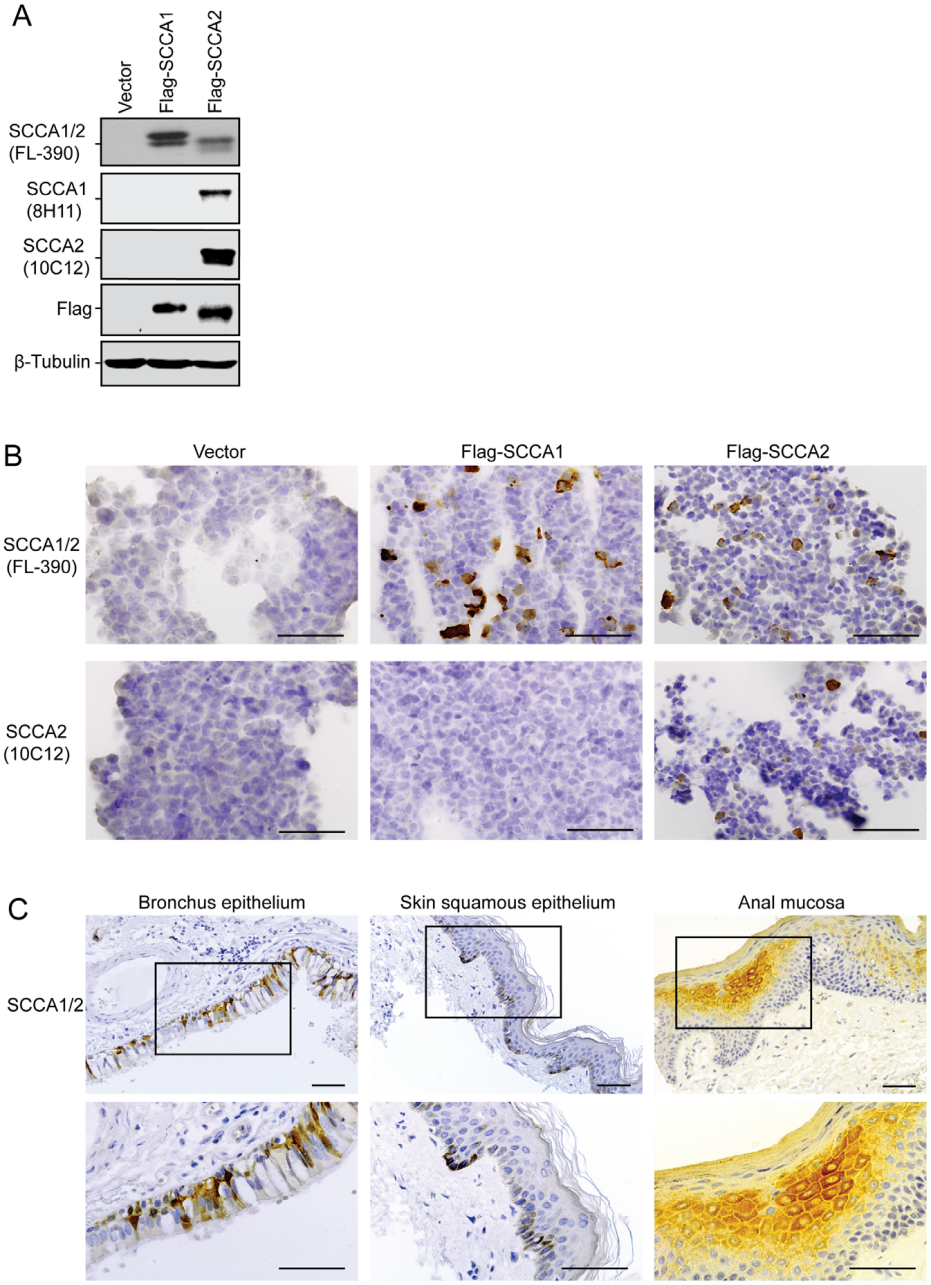
Validation of SCCA antibodies. 293T cells were transfected with either vector alone, Flag-SCCA1, or Flag-SCCA2 plasmids. (A) Cells were subjected to immunoblot analysis using three SCCA antibodies: FL-390 for SCCA1/2, 8H11 for SCCA1, and 10C12 for SCCA2, as well as Flag antibody and β-tubulin antibody. (B) Cells were fixed and embedded in paraffin. IHC was performed with FL-390 and 10C12 antibodies. (C) IHC was performed on normal human tissue using the antibody Clone FL-390. Scale bars  = 50 µm.

First, we tested three commercially available antibodies that have been previously described [7] for immunoblotting and immunohistochemistry (IHC) analysis. According to the manufacturer's instruction, one antibody is supposed to recognize both SCCA1 and SCCA2 (Santa Cruz Biotechnology, Inc. Clone FL-390), one to recognize specifically SCCA1 (Santa Cruz, Clone 8H11), and another to recognize specifically SCCA2 (Santa Cruz, Clone 10C12). We characterized these three antibodies using 293T cells transfected with Flag-SCCA1 or Flag-SCCA2. While Clone FL-390 recognized both SCCA1 and SCCA2, and Clone 10C12 specifically recognized SCCA2 as described by the manufacturer, Clone 8H11 failed to recognize SCCA1 and instead recognized SCCA2 (Fig. 1A). The specificity of the antibodies was further examined by immunocytochemistry using paraffin-embedded 293T cells expressing Flag-SCCA1 or Flag-SCCA2. Similar to the immunoblotting analysis, Clone FL-390 recognized both SCCA1 and SCCA2, while Clone 10C12 recognized only SCCA2 (Fig. 1B). The 8H11 antibody, which was described to specifically recognize SCCA1 (Santa Cruz Biotechnology Product Information; [8]), failed to do so in our hands. These results indicate that Clone FL-390 is a reliable and more efficient antibody for recognizing both SCCA1 and SCCA2. Indeed, when FL-390 was tested on paraffin-embedded normal human tissues, it revealed SCCA expression in the ciliated pseudo-stratified columnar epithelial of the bronchus, in suprabasal and basal epidermal keratinocytes, and in the suprabasal keratinocytes of the stratified squamous epithelial of the anal mucosa (Fig. 1C), consistent with reports in literature describing SCCA expression patterns [7]. Therefore, although efforts have been reported to individually detect SCCA1 and SCCA2, as these two isoforms have distinct biological functions [8], [9], we choose to use Clone FL-390 for the subsequent immunoblotting and IHC assays, because 1) Clone FL-390 has better efficiency for both immunoblotting and IHC analysis; and 2) based on current clinical studies, an assay recognizing both SCCA1 and SCCA2 is recommended for optimal clinical sensitivity [10].

### SCCA expression is elevated in breast cancer cell lines and tissues

**Figure 2:**
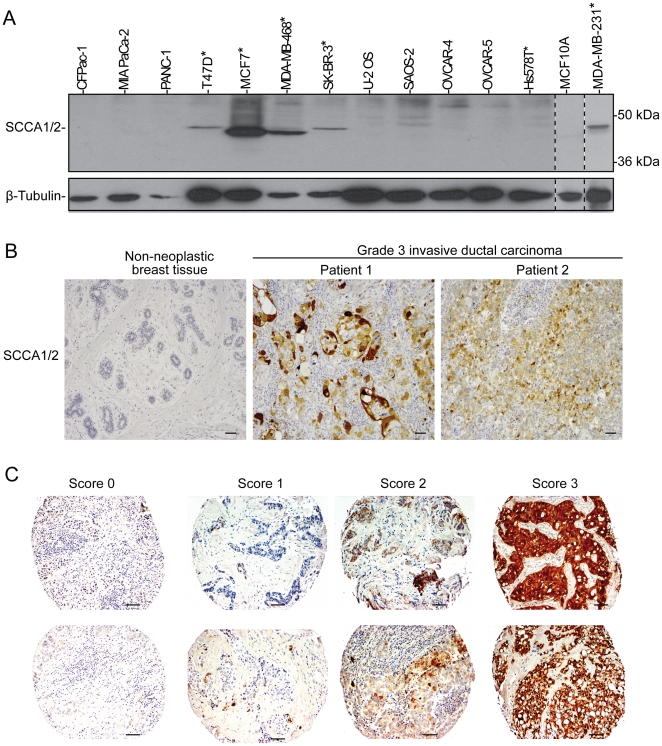
Elevated SCCA expression is found in human breast cancer cell lines and human breast cancer carcinomas, but not in normal breast epithelium. (A) A panel of human cancer cells was probed for SCCA expression by immunoblotting. Five out of the six breast cancer cell lines (denoted with an asterisk) were positive for SCCA expression. (B) IHC analysis was performed on an array of breast carcinomas and normal breast tissue obtained from CHTN, using antibody FL-390. Representative images of normal breast tissue and sections with Grade III invasive ductal carcinoma are shown. (C) IHC staining was performed on the NCI CDP breast cancer progression and the prognostic TMAs using antibody FL-390. SCCA staining was scored on a tiered-scale (0–3). A representative panel is shown. Scale bars  = 50 µm.

In searching for evidence that SCCA may be associated with human breast cancer, we first compared SCCA expression levels among a number of tumor cell lines including a non-neoplastic breast epithelial cell line MCF10A, breast cancer lines (T47D, MCF7, MDA-MB-468, SK-BR-3, Hs578T, and MDA-MB-231), pancreatic cancer lines (CFPac-1, MIA PaCa-2, PANC-1), osteosarcoma lines (U-2 OS and SAOS-2), and ovarian cancer lines (OVCAR-4 and OVCAR-5). SCCA was detected at various levels in 5 out of 6 of the breast cancer cell lines (Fig. 2A), indicating that SCCA expression is elevated in certain types of breast cancers. It remains to be determined why the bands on the immunoblots appeared to migrate differently in these breast cancer cell lines (Fig. 2A). Possible explanations include the different isoforms of SCCA or the proteolytic cleavage of SCCA [11]. Interestingly, the 5 positive cell lines (T47D, MCF7, MDA-MB-468, SK-BR-3, and MDA-MB-231) were derived from metastatic invasive ductal carcinomas [12], [13], whereas the Hs578T cell line was derived from a patient with primary carcinosarcoma [14]. Taken together, these results indicate that expression of SCCA is elevated in certain breast cancers and may correlate with invasive ductal carcinoma.

We next examined the expression of SCCA in human breast cancer tissues. A breast carcinoma progression tissue microarray (TMA) was obtained from the Cooperative Human Tissue Network (CHTN) at the University of Virginia. This TMA contained 7 cases of non-neoplastic breast epithelium from healthy subjects, 7 cases of non-neoplastic breast epithelium from subjects with breast cancer, and 42 cases ranging from low grade ductal carcinoma in situ (DCIS) to metastatic breast carcinoma. IHC was performed with Clone FL-390. Immunohistochemical staining was semi-quantitatively scored on a tiered scale (0-3) as a percentage of positive tumor cells (Fig. 2C). The expression levels were recorded as percent of tumor cells with SCCA expression 0 (no positive staining), 1 (positive staining in less than 10% of cells), 2 (positive staining in 10-50% of cells), and 3 (positive staining in over 50% cells). Tumor tissues with a score higher than 1 were counted positive for SCCA expression. Elevated expression of SCCA was detected in 4 high grade and one low grade tumors, and was not present in normal breast tissue (Fig. 2B). These results indicate that SCCA expression is elevated in certain breast cancers.

### SCCA expression in breast carcinomas correlates to both grade and stage

To further investigate the involvement of SCCA in breast cancer, we obtained two large scale TMA case sets from the NCI Cancer Diagnosis Program (CDP). One is the 2nd generation breast cancer progression TMA, and the other is the CDP 2008 breast cancer prognostic TMA that contains three non-metastatic TNM stages I-III as defined by AJCC Manual for Staging of Cancer [15]. SCCA expression was detected by IHC using Clone FL-390. In the NCI CDP progression TMA, IHC analysis revealed that all of the normal breast tissue specimens were negative for SCCA expression. When compared with the grading information provided for the 291 cases, SCCA was found in only Grade II (n = 5) and Grade III (n = 8) specimens (p = 0.016) (Table 1). These results indicate that elevated SCCA expression is associated with breast carcinomas but not normal breast epithelium, and this expression correlates with the grade of the invasive cancer.

**Table 1:** SCCA expression correlates to high grade breast cancer in the CDP progression TMA.

	Case Set 3	Case Set 5	Case Set 7	Total
Normal tissue(SCCA Positive)	23(0)	23(0)	23(0)	**69(0)**
Grade 1(SCCA Positive)	18(0)	9(0)	9(0)	**36(0)**
Grade 2(SCCA Positive)	34(2)	34(2)	36(1)	**104(5)**
Grade 3(SCCA Positive)	21(2)	33(3)	28(3)	**82(8)**
				p = 0.016

The three CDP progression TMA case sets (designated as Case Sets 3, 5, and 7 by CDP) were probed for SCCA expression and scored. The grading information was provided by CDP in 291 accountable samples.

In the prognostic TMA case sets, SCCA expression was also detected in primarily Grade II and Grade III tissue specimens, only 1 Grade I sample screened positive (p<0.0001) (Table 2). Strikingly, 68.5% of the positive specimens corresponded with Grade III breast carcinoma even though Grade III specimens were underrepresented in these TMA case sets (only 28.9% of the total breast carcinoma tissue samples). Cumulatively, among the CDP progression (Table 1) and the prognostic (Table 2) TMAs, all 124 non-neoplastic specimens were found to be SCCA negative, whereas 1 of the 330 (0.3%) Grade I cases, 16 of the 638 (2.5%) Grade II cases and 37 of the 392 (9.4%) Grade III cases were SCCA-positive *(*p<0.0001) (Table 3). Importantly, SCCA positivity also correlated with stage of the disease, as 8.6% of Stage III tissue samples tested positive for SCCA expression, whereas only 2.4% and 3.1% of Stage I and II samples, respectively, showed SCCA expression (p = 0.0005) (Table 4). In addition, our study encompassed 1,138 breast cancer samples with ductal/lobular classification. While 40 of the 1,029 ductal carcinoma specimens were SCCA-positive, only 1 SCCA-positive case was found in the 109 lobular carcinoma specimens.

**Table 2:** SCCA expression correlates to high grade breast carcinoma in the CDP prognostic TMA.

	Case Sets 9–13 (Stage I)	Case Sets 14–17 (Stage II)	Case Sets 18–19 (Stage III)	Total
Normal tissue(SCCA Positive)	25(0)	20(0)	10(0)	**55(0)**
Grade 1(SCCA Positive)	197(0)	73(0)	24(1)	**294(1)**
Grade 2(SCCA Positive)	268(4)	175(1)	91(6)	**534(11)**
Grade 3(SCCA Positive)	108(10)	143(11)	59(8)	**310(29)**
				p<0.0001

The Stage I (Case Sets 9–13), Stage II (Case Sets 14–17), and Stage III (Case Sets 18–19) prognostic TMAs were obtained from CDP, containing 598, 411, and 184 tissue specimens, respectively. IHC was performed against SCCA. The tissue was scored and the SCCA-positive cases are shown in parentheses against the number of cases in each grade. Note that all of the normal tissue were SCCA-negative, whereas 1 out of 294 Grade I, 11 out of the 534 Grade II, and 29 out of 310 Grade III cases were SCCA-positive.

**Table 3:** SCCA expression correlates to high grade breast carcinomas.

	# SCCA Positive/Total Breast Carcinoma Specimen
Normal/Non-Neoplastic	0/124
Grade I	1/330(0.3%)
Grade II	16/638 (2.5%)
Grade III	37/392 (9.4%)
	p<0.0001

The cumulative results of the SCCA positivity against the breast carcinoma grading from the CDP progression and prognostic TMAs are shown. SCCA is negative in the 124 normal or non-neoplastic cases of breast tissue. SCCA is positive in 1 of the 330 (0.3%) Grade I cases, 16 of the 638 (2.5%) Grade II cases, and in 37 of the 392 (9.4%) Grade III cases.

**Table 4:** SCCA expression correlates to advanced stage breast carcinomas.

	# SCCA Positive/Total Breast Carcinoma Specimen
Normal/Non-Neoplastic	0/55
Stage I	14/573 (2.4%)
Stage II	12/391 (3.1%)
Stage III	15/174 (8.6%)
	p = 0.0005

The Stage I, Stage II, and Stage III prognostic TMAs were obtained from CDP. IHC was against SCCA. The tissue was scored and the SCCA-positive cases are shown in parentheses against the number of cases for each stage. Note that all of the normal tissue were SCCA-negative, whereas 14 out of 573 (2.4%) Stage I, 12 out of the 391 (3.1%) Stage II, and 15 out of 174 (8.6%) Stage III cases were SCCA-positive.

Moreover, while no statistical difference was detected for the mean age of diagnosis (p = 0.38), the mean size of tumors was 2.41 cm for SCCA-negative versus 3.58 cm for SCCA-positive tumors (p<0.0001). The progression TMA also came with information for the expression of estrogen receptor (ER) and progesterone receptor (PR), but not Her2/neu status. Out of the 13 SCCA positive breast carcinoma specimens, 9 cases (69%) were classified as double negative (DN) for the expression of both ER and PR, whereas only 24% of the SCCA-negative tumor specimens were DN (p = 0.0009). This is consistent with the notion that tumors negative for both hormone receptors are more likely to be Grade III and to have a larger mean tumor size [16]. Taken together, the IHC results obtained from both the progression and prognostic TMAs indicate that SCCA expression correlates to high grade and high stage breast carcinomas.

### SCCA correlates with decreased chance of overall survival and recurrence-free survival

**Figure 3:**
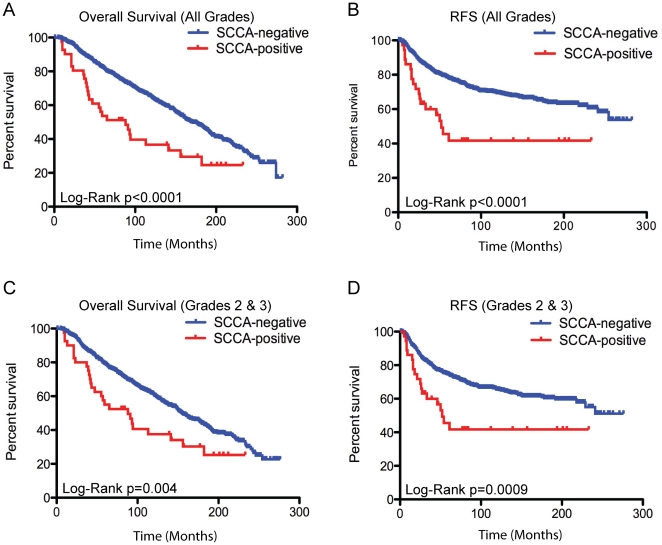
SCCA expression correlates to a decreased overall survival and recurrence-free survival. IHC was performed using the SCCA antibody FL-390 on the CDP prognostic TMAs. Kaplan-Meier survival curves for all patients (A–B) and only Grade II and Grade III patients (C–D) with SCCA-positive and negative tumors were compared for overall survival (A&C) and recurrence-free survival (B&D).

Following the proposal by Hudis *et al* [17], survival analyses were carried out to compare both overall survival (OS) and recurrence-free survival (RFS) using the information included with the prognostic TMAs. SCCA positivity correlated with decreased OS and RFS (Fig. 3A–B) (OS; hazard ratio (HR), 2.75; 95% CI, 1.62–4.68; Log-rank p = 0.0002) (RFS: HR, 4.64; 95% CI, 2.26–9.55; Log-rank p<0.0001). Furthermore, comparing only Grade II and III breast cancers, patients with SCCA expression had a decreased OS (HR, 2.08; 95% CI, 1.26–3.44; Log-rank = 0.004). The median OS was 155.0 months for SCCA-negative patients, with a 5-year survival rate of 79.1%, whereas the median OS was 88.0 months, with a 5-year survival rate of 54.2% for patients positive for SCCA (Fig. 3C). SCCA expression also correlated to a worse RFS (HR, 3.08; 95% CI, 1.59–5.98; Log-rank p = 0.0009). Grade II and III SCCA-negative patients had a 5-year RFS of 74.4%, while SCCA-positive patients had a 5-year RFS of 42.2% and a median time to recurrence of 52 months (Fig. 3D). These results further support that SCCA expression correlates with high grade breast cancer with poorer outcome.

## Discussion

SCCA was originally identified as a serological marker in cervical carcinoma [1]. Studies have shown that the SCCA levels in the serum of cervical squamous cell carcinoma patients correlates with the severity of the cancer [18]. SCCA levels have also been shown to coincide to a degree with tumor infiltration and frequency of lymph node metastasis in both cervical and esophageal squamous cell carcinomas [5], [19], [20]. Here, our study shows for the first time that elevated expression of SCCA is associated with both high grade and advanced stage human breast carcinomas. An accurate portrayal of the entire tumor may not be represented in our study due to the nature of TMAs with regard to the small tissue specimen size. Therefore we speculate that the percentage of breast cancers with SCCA positivity may have been under estimated in our study. Whole section biopsy samples will help to more accurately assess the association of SCCA with breast cancer. Similar reason may explain why an earlier study using laser microdissection and microarray analysis did not indicate any difference in SCCA expression between normal breast tissue, ductal carcinomas, and lobular carcinomas, as only 5 invasive ductal carcinomas and 5 invasive lobular carcinomas were examined [21], and according to our study, SCCA positive tumors represented only about 4% of breast cancer specimens.

In addition to the correlation between SCCA expression and high grade and advanced stage breast carcinoma, we find that those with SCCA-positive tumors have a worse clinical outcome, including decreased OS and RFS. Our results are consistent with other studies that have shown high levels of SCCA correlate to disease recurrence [22]. Additionally, SCCA serum levels have been regarded as a valuable marker of patient response to both radiation and chemotherapy [18], [23]. While our study links SCCA tissue expression levels with disease state, overall survival and recurrence, it hints at the possibility of utilizing SCCA serum levels in breast cancer patients to determine clinical outcome and response to therapy.

A number of reports have shown that breast SCC is an aggressive disease behaving like poorly differentiated breast adenocarcinoma [24], [25]. Our finding that SCCA positivity is associated with high grade breast carcinoma is consistent with this notion. In addition, the features of the SCCA-positive cases we identified in this study are very similar to those obtained from a study carried out at M.D. Anderson Cancer Center, where 33 breast SCC patients identified from 1985–2001 were analyzed and shown to correlate with worse OS and RFS [26]. Hence, SCCA expression may be indicative of breast SCC and has the potential to be a valuable marker of breast SCC, as well as predicting disease severity and response to treatment as has been demonstrated in other SCCs.

Despite its clear clinical relevance, the biological function of SCCA remains unclear. In some cancers, increased expression of Serpins is likely to protect tumor cells by guarding against lysosomal damage, release of toxic proteases such as cathepsins, and subsequent cell death. On the other hand, elevated SCCA may compromise the cell's ability to degrade misfolded proteins, and render tumor cells more vulnerable to treatment that causes proteotoxicity. Consistent with the correlation between SCCA1 expression and high grade breast carcinoma, we have found that ectopically expressed SCCA1 can induce de-differentiation of MCF10A non-neoplastic breast cell line and promote transformation both in cell culture and in vivo (Sheshadri et al, unpublished data). It is also interesting to note that while we detected elevated SCCA expression in about 4% breast cancer specimens, 5 out of the 6 breast cancer cell lines were positive for SCCA expression (Fig. 2A). This suggests that SCCA-expressing cells may better adapt to cell culture hence have a survival advantage under stressed conditions. Hence, identifying SCCA in breast cancer may provide a novel diagnostic approach that will help to understand the initiation and advancement of breast cancer and provide new therapeutic options.

## Materials and Methods

### Cell lines, culture, and transfection

T47D, MCF7, MDA-MB-468, SK-BR-3, MDA-MB-231, MCF10A, CFPac-1, MIA PaCa-2, SAOS-2, U-2 OS, PANC-1, and 293T cell lines were purchased from ATTC. Hs578T, OVCAR-4, and OVCAR-5 were purchased from the Division of Cancer Treatment and Diagnosis at National Cancer Institute. All cells were cultured according to ATCC or NCI recommendations supplemented with 100 units/ml penicillin and 100 μg/ml streptomycin (Invitrogen). 293T cells were transfected by Lipofectamine 2000 (Invitrogen).

### Plasmids

Human SCCA1 was cloned by RT-PCR from total RNA of MDA-MB-468 cells. Human SCCA2 was cloned by RT-PCR from total RNA of MCF 10A cells. Primers used for cloning of both SCCA1 and SCCA2: Forward primer, contains BamHI restriction site and Flag tag: 5′-CGGGATCCATGGACTACAAGGACGACGATGACAAGACCATGAATTCACTCAGTGAAGCC-3′. Reverse primer: contains XhoI restriction site: 5′-CCCTCGAGCATCTACGGG GATGAGAATCTGCCA-3′. RT-PCR products were ligated into the pCR2.1-TOPO vector (Invitrogen) and verified through sequencing (Stony Brook University DNA Sequencing Facility). Sequences were verified against reported sequences at NCBI GenBank. Both SCCA1 and SCCA2 were then subcloned into the LPC retroviral expression vector.

### Immunoblot analysis

Cell lysates were prepared in RIPA buffer (1% Sodium Deoxycholate, 0.1% SDS, 1% Triton X-100, 0.01 M Tris pH 8.0, 0.14 M NaCl). Protein expression was examined by western blotting using SCCA1/2 (Santa Cruz, FL-390), SCCA1 (Santa Cruz, 8H11), SCCA2 (Santa Cruz, 10C12), FLAG (M2, Sigma), and β-tubulin (Sigma). All primary antibodies were incubated overnight at 4°C. Horseradish peroxidase-conjugated goat anti-rabbit (Rockland) or goat anti-mouse (Rockland) antibodies were used as secondary antibodies. Western blots were developed using an ECL detection kit (Thermo Scientific).

### Tissue microarrays

The tissue microarrays (TMAs) used in this study were obtained from both the Cooperative Human Tissue Network (CHTN) at the University of Virginia and the National Cancer Institute (NCI) Cancer Diagnosis Program (CDP). The TMA obtained from the CHTN contained 7 cases of non-neoplastic breast tissue from healthy subjects, 7 cases of non-neoplastic breast tissue from subjects with breast cancer, and 42 cases ranging from low grade DCIS to metastatic breast carcinoma. The progression TMA obtained from the CDP (Sets 3, 5, and 7) were designed by NCI statisticians to provide high statistical power and are suitable for use in the investigation of differences in the prevalence of potential markers in invasive breast cancer. Each TMA consists of 288 cores (0.6 mm) taken from paraffin-embedded specimens that represent a total of 252 breast cancer and normal breast tissue specimens plus 36 controls. The prognostic TMA obtained from the CDP (Stage I, Sets 9–13; Stage II, Sets 14–17; Stage III, Sets 18–19) are designed to examine potential prognostic markers in non-metastatic breast cancer. Each TMA consists of between 100–150 cores, including 100–120 breast cancer and normal breast specimens plus 5–20 control cores.

### Immunohistochemistry

Paraffin-embedded TMA arrays were deparaffinized and rehydrated with graded ethanol. Endogenous peroxidase was blocked using 3% hydrogen peroxide. Antigen retrieval was accomplished using 10 mM citrate buffer (pH 6.0). The sections were blocked with 5% goat serum for one hour at room temperature. SCCA primary antibody (FL-390, 1:200; 10C12, 1:50) was diluted in the blocking solution. Slides were incubated with the primary antibody overnight at 4°C. Slides were then washed and incubated with the appropriate biotinylated secondary antibody (1:1,000) for one hour at room temperature. Following a wash series, tissue was incubated with avidin/biotinylated HRP (ABC Elite kit from Vector Labs) according to manufacturer's instructions. Slides were submerged in diaminobenzidine (DAB)/H_2_O_2_ substrate solution until the desired staining intensity was obtained and slides were counterstained with hematoxylin. Slides were observed and photographs taken using an Olympus BX41 microscope.

### TMA analysis

Damaged core spots and those that were entirely adipose tissue were eliminated from scoring. The sections were scored independently by two evaluators blinded to the clinical status of the patients. The results were classified as percent of tumor cells with SCCA expression: 0, no expression; 1, <10%; 2, 10–50%; 3, >50%. Clinical data was then used to correlate SCCA expression with various clinicopathological variables.

### Statistical analysis

Two sample t-tests were used to compare continuous clinical features, such as age and size of invasive tumor between SCCA-negative and positive samples; Chi-squared tests and Fischer's exact tests, when applicable, were used to assess statistical significance of various categorical clinical features between SCCA-negative and SCCA-positive samples. Kaplan-Meier curves for overall survival (OS) and recurrence-free survival (RFS) were constructed for SCCA-negative and positive patients and compared using the Log-rank (Mantel-Cox) test. Hazard ratios and their 95% confidence intervals were derived, together with median survival times and 5-year survival rates. Statistical analyses were performed with GraphPad Prism (Graphpad Software Inc). Two-sided P values <0.05 were considered statistically significant.
